# New In Vitro Model of Oxidative Stress: Human Prostate Cells Injured with 2,2-diphenyl-1-picrylhydrazyl (DPPH) for the Screening of Antioxidants

**DOI:** 10.3390/ijms21228707

**Published:** 2020-11-18

**Authors:** Christian Galasso, Concetta Piscitelli, Christophe Brunet, Clementina Sansone

**Affiliations:** Marine Biotechnology Department, Stazione Zoologica Anton Dohrn, Istituto Nazionale di Biologia, Ecologia e Biotecnologie Marine, Villa Comunale, 80121 Napoli, Italy; concetta.piscitelli@szn.it (C.P.); christophe.brunet@szn.it (C.B.); clementina.sansone@szn.it (C.S.)

**Keywords:** DPPH, oxidative stress, ROS, natural products, *Tetraselmis suecica*, PNT2, cell-based antioxidant method, in vitro antioxidant screening

## Abstract

The antioxidant activity of natural compounds consists in their ability to modulate gene and protein expression, thus inducing an integrated cell protective response and repair processes against oxidative stress. New screening tools and methodologies are crucial for the actual requirement of new products with antioxidant activity to boost endogenous oxidative stress responsive pathways, Reactive Oxygen Species (ROS) metabolism and immune system activity, preserving human health and wellness. In this study, we performed and tested an integrated oxidative stress analysis, using DPPH assay and PNT2 cells injured with DPPH. We firstly investigated the mechanism of action of the oxidising agent (DPPH) on PNT2 cells, studying the variation in cell viability, oxidative stress genes, inflammatory mediator and ROS levels. The results reveal that DPPH activated ROS production and release of Prostaglandin E_2_ in PNT2 at low and intermediate doses, while cells switched from survival to cell death signals at high doses of the oxidising agent. This new in vitro oxidative stress model was validated by using Trolox, β-carotene and total extract of the green microalga *Testraselmis suecica*. Only the *T*. *suecica* extract can completely counteract DPPH-induced injury, since its chemical complexity demonstrated a multilevel protecting and neutralising effect against oxidative stress in PNT2.

## 1. Introduction

The oxidative stress defense includes a complex intracellular system of enzymatic and non-enzymatic pathways that are able to inhibit the harmful effects of free radicals and other oxidants. Superoxide radical (O_2_•^−^), hydrogen peroxide (H_2_O_2_) and hydroxyl radical (HO•), not being all free radicals, were called collectively Reactive Oxygen Species (ROS) due to their higher reactivity than molecular oxygen [1]. ROS are generated during aerobic metabolism processes and are able to regulate many pathways by directly reacting with proteins and by modulating transcription factors and gene expression [2]. Thus, ROS are involved in the regulation of many biological mechanisms, such as autophagy, cell proliferation, differentiation, migration, immunity, longevity as well as cellular stress response [3].

Uncontrolled ROS levels can damage crucial cellular components, such as DNA, proteins and lipids and can induce irreversible damages to organelles and structures. Consequently, cells undergo programmed cell death (PCD) [4], inducing in some cases chronic perturbation of the tissue microenvironment. In order to avoid irreversible cell damage and PCD induced by ROS injury, living organisms possess finely regulated intracellular signaling pathways controlled by a mitochondrial system [5]. Indeed, cells activate detoxifying pathways able to scavenge and inhibit the harmful effects of free radicals and other oxidizing agents. Among antioxidant enzymes, the superoxide dismutases (reduce O_2_•^−^ to O_2_ and H_2_O_2_), catalases and glutathione peroxidases (reduce H_2_O_2_ to H_2_O) are frequently activated.

Although highly efficient, the endogenous antioxidant defense system often requires exogenous antioxidants to optimize a high protection from oxidative stress [6,7,8], which is the reason why the discovery of new natural antioxidants has sparked increasing interest [7]. Intake of exogenous antioxidants can improve the quality of life by preventing or postponing the onset of degenerative diseases, related to deleterious effects of chronic oxidative stress status in the human body [9,10]. In order to assess the antioxidant properties of new natural compounds, various antioxidant assays were developed. Assays currently used consist in different methodologies, belonging to hydrogen atom transfer (HAT) or single electron transfer (SET) principles [11,12]. The HAT assays measure the capacity of an antioxidant to quench free radicals by hydrogen donation; an example is the oxygen radical absorbance capacity (ORAC) assay. Instead, the SET assays evaluate the capacity of a potential antioxidant to transfer one electron to a compound. This second type of assays includes the 2, 2-diphenyl-1-picrylhydrazyl (DPPH) assay. The DPPH method is one of the most common antioxidant assays, allowing for the evaluation of the radical scavenging activity of a large group of samples simultaneously, based on its simplicity, accuracy, speed, and low cost [13]. However, these kinds of assays focus on the radical quenching capacity of the compounds, without any evaluation of their potential ability to modulate gene and protein expression, to enhance endogenous antioxidant enzymes function and/or trigger specific cell signaling aiming the cells to architect an integrated protective response against oxidative stress [14,15]. To cope with this experimental failing, a combined approach of cell- and biochemical-based analysis is required to fully characterize the antioxidant effect of compounds [16]. Nowadays, cell-based analysis is relied on the assessment of protective effect of compounds or total extracts against H_2_O_2_-induced cytotoxicity in different human cells [17]. H_2_O_2_ intracellular accumulation induces oxidative stress and inflammation; this oxidizing agent is often correlated with the onset and development of various diseases, such as cancer, diabetes, cardiovascular diseases [18,19,20]. Indeed, H_2_O_2_ is frequently used in viability assay and cell signaling studies to evaluate the protective or repair effects of natural products against H_2_O_2_-induced cell damage [21].

The aim of the present work is to define an innovative and integrated methodological approach, from biochemical assessment of potential antioxidant compounds to in vitro stimulation of defense responses on human cells against oxidative stress. Here, we investigated, for the first time, the effect of DPPH on the normal human prostate cells (PNT2), integrating cell viability changes, ROS production, as well as variations of gene expression and proteins release. We described dose–response relationship helpful to reproduce and standardize this oxidative stress cell-based in vitro model, which can be used for testing the antioxidant capacity of new natural extracts or compounds. Moreover, a combination of DPPH colorimetric assay with the cell-based (DPPH-PNT2) method was tested for the validation of a multilevel experimental procedure to assess radical scavenging/antioxidant activity of natural compounds/extracts. The effectiveness of this experimental combined approach was verified using two pure compounds (Trolox and β-carotene). Trolox is known to scavenge free radicals in order to avoid lipid peroxidation, and to protect cells against oxidative stress [22,23]. β-carotene is an antioxidant but which negatively responds to DPPH radical scavenging assay (as all carotenes, [24]). In addition, we used a total extract to verify the response of this cell-based oxidative model; *T*. *suecica* extract was selected, since it previously demonstrated having a strong scavenge and repair effect on human cells [21].

## 2. Results

### 2.1. Cell Viability

**Figure 1:**
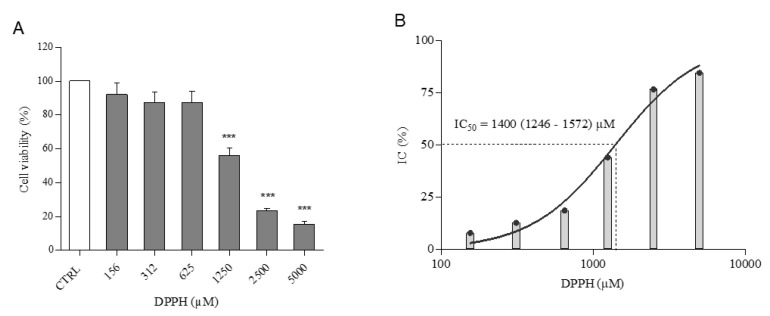
Cell viability of PNT2 treated with DPPH. (**A**) Dose–response curve of DPPH on cell viability of PNT2. Cells were treated with six concentrations of DPPH (156, 312, 625, 1250, 2500 and 5000 µM) for 24 h. The results are expressed as percent of cell viability calculated as the ratio between mean absorbance of each treatment and mean absorbance of control. (**B**) Inhibitory Concentration (IC) of viability data, expressed as a percentage. All values are represented as the mean ± SD of three independent experiments. Asterisks indicate the statistically significant difference compared to a negative control (*** *p* ≤ 0.001; Dunnett’s test).

Six concentrations of DPPH were tested for 24 h on PNT2 cells (Figure 1A). results showed that 625 µM and the lower concentrations of DPPH did not exert significant cytotoxic effect on cells. Conversely, DPPH, at 1250, 2500 and 5000 µM, induced a reduction of viable cells in a dose-dependent manner (56, 23 and 15%, respectively). DPPH showed an IC_50_ of 1400 µM, with a 95% confidence interval (calculated for values not normally distributed) between 1246 and 1572 µM (Figure 1B).

### 2.2. Gene Expression

**Figure 2:**
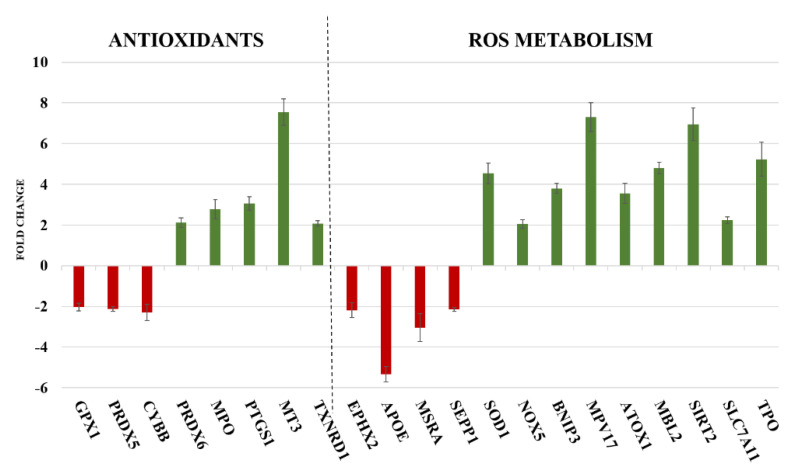
Up− and down−regulation of genes after DPPH treatment (1250 µM for 2 h) on PNT2 cells. Gene expression was evaluated on RNA extracted from three different biological replicates; histograms and error bars represent mean ± SD. Expression values greater or lower than ± 2.00 fold change were considered significant. Genes were graphically divided into two groups: genes with a key role in endogenous antioxidant mechanisms (left panel); genes encoding for proteins involved in Reactive Oxygen Species (ROS) metabolism (right panel).

**Figure 3:**
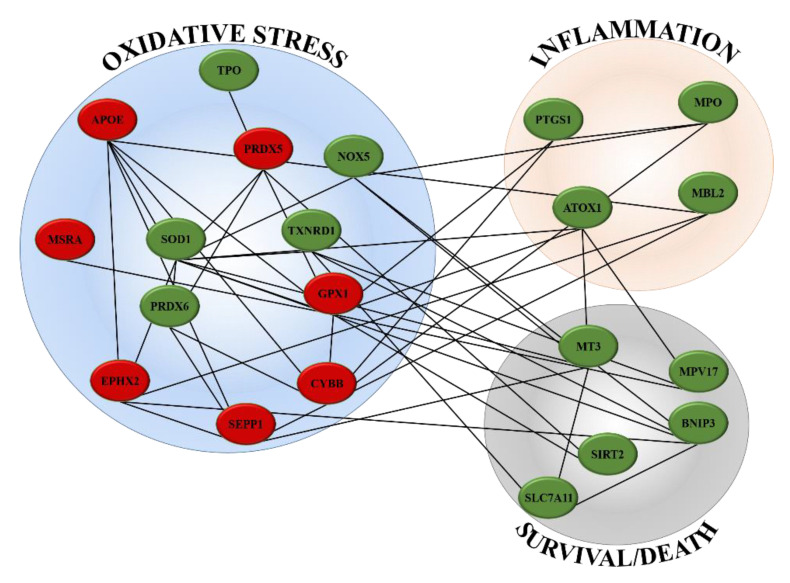
In silico prediction of interaction network of differentially expressed genes. The network was created using the STRING database and GeneMANIA web site (green nodes represent up-regulated genes and red nodes represent down-regulated genes).

Among the 84 oxidative stress-associated genes tested, DPPH induced variation of gene expression in 21 of them, that is, the 25% of genes investigated (Table S1 reports the list of all genes analysed and Table S2 reports fold change values of significantly variated oxidative stress-associated genes; see Supplementary File). This analysis examined peroxidases genes family, including glutathione peroxidases (GPx) and peroxiredoxins (TPx). The genes involved in reactive oxygen species (ROS) metabolism, such as oxidative stress responsive genes and those involved in superoxide metabolism such as superoxide dismutases (SOD) were also included in this study (Figure 2). Belonging to the endogenous antioxidants superfamily, GPX1, PRDX5 and CYBB were found down-regulated (−2.03, -2.12 and -2.29 fold change, respectively), while PRDX6, MPO, PTGS1, MT3 and TXNRD1 were up-regulated by DPPH (2.12, 2.77, 3.07, 7.95 and 2.08 fold change, respectively). Among ROS metabolism-related genes, DPPH induced the down-regulation of EPHX2, APOE, MSRA and SEPP1 (-2.19, -5.33, -3.05 and -2.15 fold change, respectively) and the up-regulation of SOD1, NOX5, BNIP3, MPV17, ATOX1, MBL2, SIRT2, SLC7A11 and TPO (4.54, 2.04, 3.80, 7.31, 3.56, 4.80, 6.95, 2.24 and 5.24 fold change, respectively). Using online available databases, such as STRING and GeneMANIA, a network of all genes significantly up- or down-regulated has been created (Figure 3). Genes were clustered in three groups depending on the pathways they are mainly involved: oxidative stress, inflammatory processes and control of survival/death signal.

### 2.3. ROS Levels

**Figure 4:**
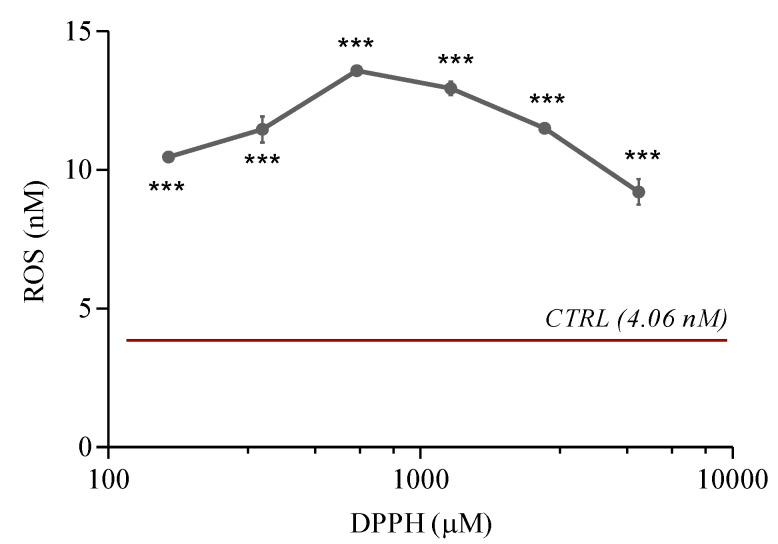
ROS concentration (expressed in nM) in PNT2 cells treated with DPPH. Cells were treated with 156, 312, 625, 1250, 2500 and 5000 µM for 6 h and the resulting cell lysate was analysed with a plate reader (Ex: 480 nm, Em: 530 nm). The red bar represents the ROS basal level in untreated PNT2 (control, CTRL). The values reported are the mean ± SD of three independent experiments. Asterisks indicate the statistically significant difference compared to the control (*** *p* ≤ 0.001; Dunnett’s test).

The treatment of PNT2 cells with the six concentrations of DPPH induced an increment of ROS generation, which was quantified. The dose–response curve of ROS production to DPPH was characterised by a maximum at 625 µM, with decreasing ROS levels at lower and higher DPPH concentrations (Figure 4). In particular, PNT2 without any treatment exhibited the basal level of ROS (4.06 nM). ROS intracellular production increased with 156, 321 and 625 µM of DPPH (10.47, 11.46 and 13.48 nM of ROS, respectively). At higher concentrations tested (i.e., 1250, 2500 and 5000 nM of DPPH), ROS levels diminished with respect to the peak observed at 625 µM (12.94, 11.51 and 9.21 nM of ROS, respectively).

### 2.4. Prostaglandin E_2_ Levels

**Figure 5:**
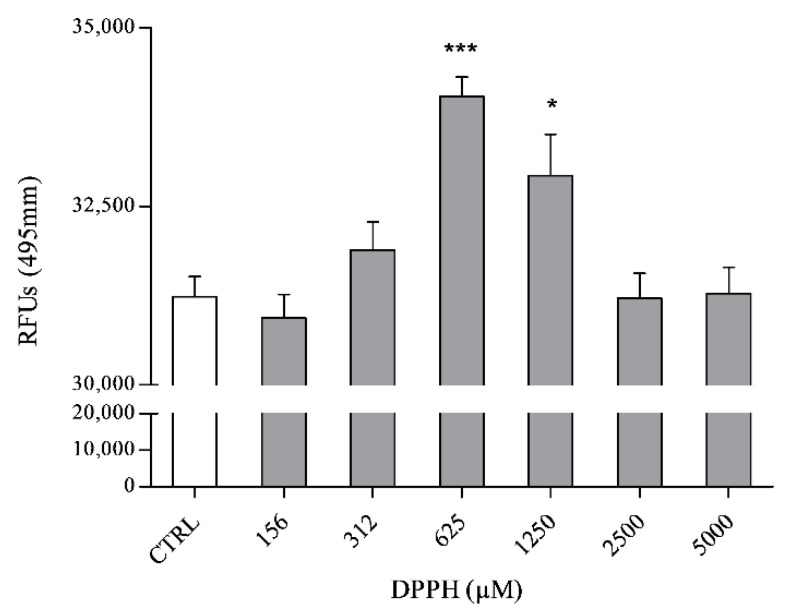
ELISA assay for the assessment of Prostaglandin E_2_ serum-release induced in PNT2 cells by six DPPH concentrations (156, 312, 625, 1250, 2500 and 5000 μM). Values are expressed as relative fluorescent units (RFUs) normalized by the protein concentration of total cell lysate and represented as the mean ± SD of PGE_2_ concentrations. Asterisks indicate statistically significant differences compared to the control (* *p* ≤ 0.05, *** *p* ≤ 0.001; Dunnett’s test).

The up-regulation of the PTGS1 gene suggests an activation of prostaglandins synthesis, such as prostaglandin E_2_ (PGE_2_). ELISA assay was used to study the release of PGE_2_ in PNT2 medium treated with the six concentrations of DPPH. Figure 5 shows the dose–response curve of the PGE_2_ release, with a peak at 625 µM. DPPH, at 312 and 1250 µM, induced lower levels of PGE_2_, with respect to 625 µM. On the contrary, cells treated with the lowest (156 µM) and highest (2500 and 5000 µM) DPPH concentrations exhibited the same PGE_2_ level as the untreated cells (control).

### 2.5. Radical Scavenging Capacity

**Figure 6:**
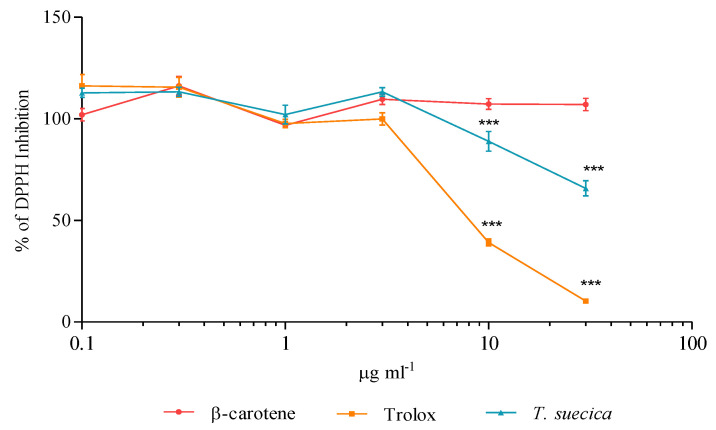
Radical scavenging capacity of β-carotene, Trolox and *Tetraselmis suecica* extract (0.1, 0.3, 1, 3, 10 and 30 µg mL^−1^) on DPPH free radical. Values are expressed as the mean ± SD of the percentage of DPPH inhibition. Asterisks indicate statistically significant differences compared to negative control (*** *p* ≤ 0.001; Dunnett’s test).

Trolox exhibited marked reducing activity toward radical species when the DPPH radical scavenging capacity was tested. The Trolox active concentrations were 10 and 30 μg mL^−1^, which resulted in a significant reduction of DPPH free radical (60.9 and 89.7%, respectively). *T*. *suecica* extract showed a radical scavenging activity at same concentrations observed for Trolox; 10 μg mL^−1^ of the extract induced 11.2% of inhibition, while 30 μg mL^−1^ caused 34.3% of inhibition. On the contrary, β-carotene did not possess significant radical scavenging activity (Figure 6).

### 2.6. In Vitro Scavenging Effect

**Figure 7:**
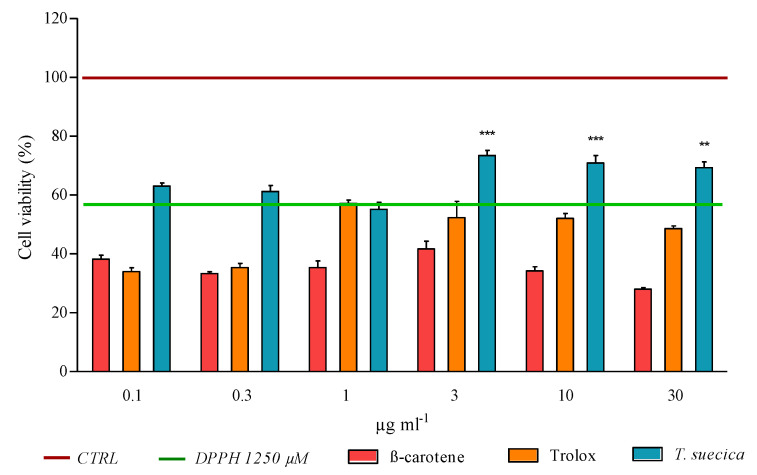
Scavenging activity of β-carotene, Trolox and *Tetraselmis suecica* extract on PNT2 cells injured with DPPH (at IC_50_ concentration, 1250 µM). Cells were pre-treated with different concentrations of β-carotene, Trolox and *T*. *suecica* extract (0.1, 0.3, 1, 3, 10 and 30 µg mL^−1^). After 1 h, DPPH was added to each well. Cell viability were determined after 24 h, by using MTT assay. The results are expressed as percent of cell viability calculated as the ratio between the mean absorbance of each treatment and the mean absorbance of the control. The red bar (at 100% of cell viability) represents untreated cells (CTRL); the green bar (at 57% of cell viability) represents PNT2 treated with only 1250 µM of DPPH (DPPH 1250 µM). All values are represented as the means ± SD of three independent experiments. Asterisks indicate the statistically significant difference compared to the positive control with DPPH (** *p* ≤ 0.01, *** *p* ≤ 0.001; Dunnett’s test).

Cell viability was also evaluated when cells were pre-treated with the three antioxidant samples and then injured with DPPH, assessing the ability of β-carotene, Trolox, and *T. suecica* extract to scavenge and protect from DPPH-induced oxidative cell damages. As shown in Figure 7, PNT2 treated only with 1250 µM of DPPH (positive control) exhibited 57% of viability after 24 h. Pre-treatment with β-carotene and Trolox did not protect cells from oxidative damage, since the two compounds induced a percentage of cell viability lower than the positive control. Conversely, *T. suecica* extract, at 3, 10 and 30 µg mL^−1^, protected PNT2 from the DPPH cytotoxic effect, showing a percentage of cell viability higher than the positive control (73, 71 and 69%, respectively). PNT2 treated with only β-Carotene, Trolox and *T. suecica* extract (at same concentrations) did not exhibit a significant reduction of viable cells (data not shown).

### 2.7. Reduction in ROS Generation by the Treatments

**Figure 8:**
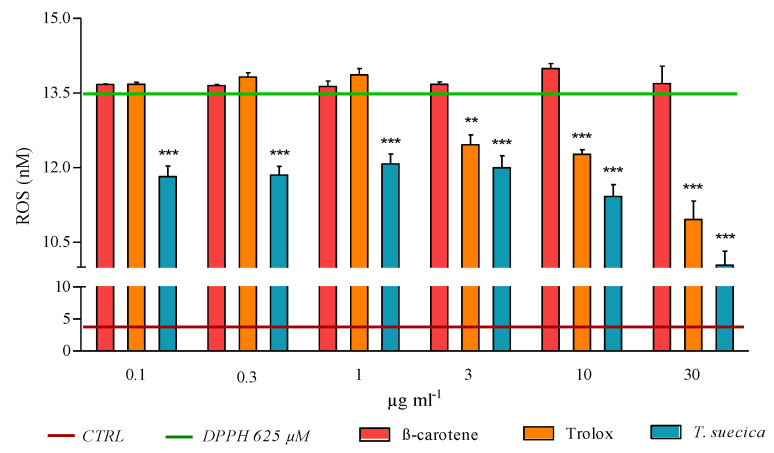
ROS concentrations in PNT2 cells pre-treated with β-carotene, Trolox and *T. suecica* extract (at 0.1, 0.3, 1, 3, 10 and 30 µg mL^−1^) and then injured with 625 µM of DPPH. After 6 h of treatment, the resulting cell lysate was analysed with a plate reader (Ex: 480 nm, Em: 530 nm). The green bar (at 13.58 nM) represents ROS levels in PNT2 cells treated only with DPPH (DPPH 625 µM); the red bar (4.06 nM) represents ROS levels in untreated cells (control, CTRL). The values reported are the mean ± SD of three independent experiments. Asterisks indicate statistically significant differences compared to positive control (** *p* < 0.01, *** *p* ≤ 0.001; Dunnett’s test).

The ability of the three samples to contrast the ROS production on PNT2 cells has been evaluated. PNT2 cells were pre-incubated with the six concentrations of β-carotene, Trolox and *T. suecica* extract (0.1, 0.3, 1, 3, 10 and 30 µg mL^−1^) and, after 1 h, treated with DPPH (at 625 µM, which induced highest ROS level). After 6 h of incubation, the control cells produced 4.06 nM of ROS, while the injured cells (PNT2 treated only with DPPH, positive control) showed a high ROS concentration (13.58 nM) (Figure 8). β-carotene was the only sample that did not influence ROS increment, since all concentrations tested showed ROS levels comparable to the positive control. On the contrary, Trolox at 3, 10 and 30 µg mL^−1^ reduced ROS concentrations in PNT2 cells (12.46, 12.27, and 10.96 nM, respectively). *T*. *suecica* extract induced a significant reduction in ROS levels at all concentrations tested. In particular, ROS generation decreased from 0.1 to 30 µg mL^−1^ of extract (11.82, 11.85, 12.08, 11.99, 11.42 and 10.04 nM, respectively) (Figure 8).

## 3. Discussion

Oxidative stress is one of the principal threats for human cells and tissues, inducing serious and fatal diseases [25]. Cells possess endogenous defence mechanisms to attenuate or eliminate oxidative injuries. Exogenous antioxidants can augment the antioxidant capacity of cells and tissues, reducing the risk of certain diseases [9]. For this reason, new in vitro oxidative stress models and protocols for the screening of natural antioxidants constitute a priority in modern drug discovery. With this study, we describe an integrated pipeline useful for the characterization of antioxidant activity of extracts and compounds, using a combination of biochemical assay (DPPH assay) and cell-based oxidative stress model. In particular, the new oxidative stress model is based on human prostatic epithelial cells (PNT2) and DPPH as an oxidising agent. The choice of cell line is based on evidence that prostatic tissue is one of the main targets of oxidative stress in the human body [26,27]. This is the first attempt in which DPPH was used as an oxidising agent on in vitro cell cultures. DPPH-related cell damages were characterized, at the gene and protein level, on PNT2 cells. In addition, pure compounds and a total extract were used to validate the integrated pipeline (formed by DPPH assay and the new in vitro oxidative stress model). Interestingly, DPPH induced a biphasic response in PNT2 cells. Indeed, concentrations ≤625 µM of the oxidising agent activated ROS production and induced the release of inflammatory mediators in prostatic cells; when cells were treated with higher DPPH concentrations (1250, 2500 and 5000 µM) cell death was triggered. The biphasic cell response is evident in our in vitro assays (Figure 1A, Figure 4 and Figure 5), where ROS and PGE_2_ concentrations showed a peak at 625 µM, while cell viability started to significantly decrease from 1250 µM, in a dose-dependent manner. The initial phase of cell response to not-cytotoxic DPPH concentrations (right portion of bell-shaped curves in Figure 4 and Figure 5) is characterised by a linear increment of oxidative stress and inflammatory mechanisms with a maximum at 625 µM of DPPH. At higher concentrations, DPPH provoked a significant reduction in cell viability (Figure 1A) that suggests an activation of cell death, resulting in the downstream inhibition of all other cell response pathways not involved in death mechanisms, such as oxidative stress regulation and inflammatory mediators biosynthesis (left portion of bell-shaped curves in Figure 4 and Figure 5).

Induction of oxidative stress, inflammation and cell death are also evident from in silica prediction analysis, where DPPH targeted genes involved in these three pathways (Figure 2 and Figure 3). Table 1 reports the function and role of genes found up- or downregulated in PNT2. Oxidative stress is the predominant mechanism in the early stage of cell response, involving the majority of genes investigated. Connections between oxidative stress, inflammation and cell death pathways are also evident from our gene results (Figure 3).

These results are in accordance with previous findings that treating cells with oxidising agents (e.g., hydrogen peroxide) described the activation and connection between ROS–inflammation–cell death as a biphasic response to oxidative stress-induced cytotoxicity [44,45]. In our results, the concentration peaks of ROS and PGE_2_ (induced at 625 µM of DPPH) could be responsible for the survival/death switch, since DPPH treatment was able to activate cell death signals only when oxidative stress and inflammation levels induced irreversible intracellular damages [4].

The combination of the well-known DPPH assay and the above described in vitro oxidative stress model (DPPH-injured PNT2 cells) was tested as new integrated screening tool for the study of potential antioxidant effect of compounds/extracts. Three antioxidant samples were used to validate the screening tool. Trolox is frequently used as a positive control in the DPPH assay, being a potent ROS scavenger [46]. β-carotene was chosen as an antioxidant compound without a DPPH radical scavenging capacity (as all carotenes, [24,47]). *T*. *suecica* extract was selected having demonstrated a high scavenging and repair activity on human cells [21].

DPPH assay indicated that Trolox is the most efficacious compound in reducing DPPH radical. *T*. *suecica* extract exhibited a moderate scavenging activity, while β-carotene did not exhibit inhibition of DPPH radical. This scavenging effect was also evaluated in the more complex cell-based PNT2-DPPH model. The three antioxidants were used as pre-treatment on PNT2 cells, to observe if they were able to scavenge and protect cells from the subsequent addition of DPPH. *T*. *suecica* extract was the only sample able to avoid DPPH-mediated cytotoxicity (Figure 7). The procedure followed for scavenging in vitro assay (pre-treatment with samples, removal of samples, treatment with DPPH; see Section 4.3) suggests that the protection mechanism occurs inside the cells, where a pool of pigments of *T*. *suecica* extract can neutralise DPPH molecules. Trolox and β-carotene did not protect PNT2 from deleterious effect of DPPH. DPPH, as suggested by the gene expression results, acts on a large array of pathways involved in antioxidant defence, superoxide and ROS metabolism; these pathways can lead to several intracellular damages and cell death, which represent downstream mechanisms. The opposite results observed for Trolox between DPPH assay (strong scavenging activity) and scavenging cell-based in vitro assay (no activity) were probably due to the reduced range of Trolox intracellular targets with respect to DPPH. Thus, it seems that a single compound, even if it scavenges a single radical species well (Figure 6), cannot offer large protection for all intracellular components damaged by DPPH (e.g., DNA, mitochondria, etc.). Indeed, Trolox was only efficacious in the reduction of ROS level (Figure 8); all other types of cell dysfunctions and injuries caused by DPPH were not prevented by Trolox, thus not inhibiting PNT2 cell death (Figure 7). β-carotene, probably acting to avoid lipid peroxidation, did not supply a sufficient cell protection against the DPPH injury, which can create dysfunctions in many cell structures and organelles, whereas *T*. *suecica* extract was the sample with a more powerful effect, inhibiting ROS generation at all concentrations tested and avoiding cell death; high concentrations of this extract reduced ROS generation until 57% and blocked cell death processes. This multifaceted antioxidant effect of *T*. *suecica* extract was probably due to the chemical complexity of this sample; the mixture of bioactive pigments in the extract acted with a synergistic effect on a larger group of intracellular targets and pathways with respect of a single compound (e.g., Trolox), inactivating DPPH not only on a biochemical level (DPPH assay), but also in a more complex cell-based experimental system, drastically reducing ROS production, intracellular oxidative stress and cell death.

## 4. Materials and Methods 

### 4.1. Preparation of the Samples for Treatments

2,2-diphenyl-1-picrylhydrazyl (DPPH) powder (CAS No. 1898-66-4, cat. No. 257621, Sigma-Aldrich), (±)-6-hydroxy-2,5,7,8-tetramethylchromane-2-carboxylic acid (herein referred as Trolox) powder (CAS No. 53188-07-1, cat. No. 238813, Sigma-Aldrich) and β-carotene powder (CAS No. 7235-40-7, cat. No. 22040, Sigma-Aldrich) were weighted and dissolved in dimethyl sulfoxide (DMSO, final concentration ≤ 0.5%). *Tetraselmis suecica* lyophilised biomass was purchased from Neoalgae company (bath No. 031218). Microalgal powder was resuspended in DMSO, vortexed and sonicated for three times, allowing extraction of pigments [48]. All samples were manipulated in dark conditions and prepared a few minutes before treatments. DMSO was used as a carrier and for extraction procedure, since it does not interfere with antioxidant and phytochemical analysis [49]. 

### 4.2. Scavenging Activity against DPPH Radical

Various concentrations of the two compounds and the extract were tested for the radical scavenger assay: 0.1, 0.3, 1, 3, 10 and 30 µg mL^−1^ of Trolox, β-carotene and *T*. *suecica* extract. These samples were mixed in a 96-well plate with a final concentration of DPPH of 0.1 mM in methanol, allowed to react for 30 min in the dark. The methanol solution was used as a negative control. At the end of incubation, absorbance was measured at 517 nm, using a microplate reader. The scavenging assay was performed in triplicate. The results are presented as a percentage of DPPH reduction with respect to the methanol negative control.

### 4.3. Culture and Treatments of Human Cells 

The PNT2 cell line (normal human prostate epithelium) was purchased from the Sigma Aldrich (product code: 95012613) and grown in RPMI 1640 supplemented with 10% (*v/v*) fetal bovine serum (FBS), 100 units mL^−1^ penicillin, 100 units mL^−1^ streptomycin and 2 mM of L-glutamine, in a 5% CO_2_ atmosphere at 37 °C. PNT2 at low passage numbers (within fifth) were used for all experiments. This allow minimising alterations in morphology, response to stimuli, growth rates, genes and proteins expression, occurring at high passage numbers, due to senescence processes.

PNT2 cells (2 × 10^3^ cells well^−1^) were seeded in a 96-well plates for all treatments and kept overnight for attachment. For the dose–response curve, cells were treated for 24 h with serial dilutions of DPPH: 5000, 2500, 1250, 625, 312 and 156 µM. For scavenging in vitro assay, cells were pre-treated for 1 h with 0.1, 0.3, 1, 3, 10 and 30 µg mL^−1^ of Trolox, β-carotene and *T*. *suecica* extract. After 1 h, media were removed and DPPH (at 1250 µM, concentration within the IC_50_ confidence interval) dissolved in fresh medium was added in each well and cells were incubated for other 24 h. All experiments were performed in three independent biological replicates. The DPPH dose–response curve and the scavenging in vitro assay on PNT2 were assessed with an MTT viability assay.

### 4.4. MTT Viability Assays

MTT viability assay (3-(4,5-dimethylthiazol-2-yl)-2,5-diphenyltetrazolium Bromide, Applichem) was performed after 24 h for all treatments. Briefly, PNT2 cells were incubated with 10 µl (5 mg mL^−1^) of MTT for 3 h at 37 °C with 5% CO_2_. The resulting formazan crystals produced by only viable cells were dissolved with 100 µL of isopropyl alcohol. The absorbance was recorded on a microplate reader at a wavelength of 570 nm. The results were represented as a percent of cell viability estimated as the ratio between the absorbance of each sample (treated cells) and the absorbance of the control (untreated cells).

### 4.5. RNA Extraction and Real-Time qPCR

PNT2 cells (2 × 10^6^ cells), used for RNA extraction, were seeded in 6-well plates and kept overnight for attachment. For gene expression studies, 1250 µM of DPPH (the concentration within the IC_50_ confidence interval) was chosen for the treatment. Gene expression was analysed after 2 h of treatment to observe activation of oxidative stress, inflammation and cell death pathways occurring in the early cell response to the oxidising agent. At the end of treatment, PNT2 cells were washed by adding cold Phosphate-Buffered Saline (PBS) and rocking gently. Cells were lysed directly in plates by adding 1 mL of TRIsure^™^ reagent (Bioline) and RNA was isolated according to the manufacturer’s protocol. RNA concentration and purity were assessed using the nanophotomer NanoDrop. The reverse transcription reaction was carried out with the RT^2^ first strand kit (Qiagen, cat. No. 330401). Real-Time quantitative Polymerase Chain Reaction (RT-qPCR) was performed using the Human Oxidative Stress Plus RT^2^ Profiler PCR Array (Qiagen, 384-well format, cat. n°: PAHS-065Y), comprising 84 oxidative stress-associated genes (see Table S1, in Supplementary Information). Three biological replicates were performed for the gene expression analysis. Plates were run on a ViiA7, Standard Fast PCR Cycling protocol with 10 µl reaction volumes. Cycling conditions used were set up in three stages: the first stage at 50 °C for 2 min and 95 °C for 10 min; the second stage consisted of 40 cycles at 95 °C for 15 s and 60 °C for 1 min; the last stage (melt curve) at 95 °C for 15 s, 60 °C for 1 min and 95 °C for 15 s qPCR data (Ct-values) were analysed with PCR array data analysis online software by, Qiagen [50]. All values greater or lower than 2.0-expression ratios with respect to the controls were considered significant. Control genes for real-time qPCR were actin-beta (ACTB), beta-2-microglobulin (B2M), glyceraldehyde-3-phosphate dehydrogenase (GAPGH), hypoxanthine phosphoribosyl transferase 1 (HPRT1) and ribosomal protein large P0 (RPLP0), the expression of which remained constant. The protein–protein interactions network was created for all genes found significantly up- or down-regulated. The network was drawn using the STRING database and GeneMANIA website.

### 4.6. ROS Assay

ROS intracellular levels were measured using The OxiSelect™ Intracellular ROS Assay Kit (CELL BIOLABS, INC, cat. No. STA-342), according to the manufacturing protocol. In brief, this kit is a cell-based assay for measuring ROS activity. Cells were seeded in a 96-well plate and then pre-incubated with dichloro-dihydro-fluorescein diacetate (DCFH-DA) for 1 h for all ROS assay experiments. A dose–response curve of ROS was obtained, treating cells for 6 h with 156, 312, 516, 1250, 2500 and 5000 µM of DPPH. Moreover, cells were pre-treated with Trolox, β-carotene and *T*. *suecica* extract at 0.1, 0.3, 1, 3, 10 and 30 µg mL^−1^. After 1 h, DPPH at 625 µM (concentration that induced highest ROS levels, without cytotoxicity) was added and cells were incubate for 6 h. At the end of incubation (at 37 °C, 5% CO_2_), medium was removed and lysis buffer was added to each well; fluorescence was read using microplate reader Infinite^®^ M1000 PRO (Tecan, Ex: 480 nm, Em: 530 nm). The ROS content in cells was determined by comparison with the predetermined DCF standard curve. 

### 4.7. ELISA Assay for PGE_2_

PNT2 (2 × 10^6^ cells), used for PGE_2_ assessment (Abcam, product No. ab2318), were seeded in 6-well plates and kept overnight for attachment. Cells were treated with 156, 312, 516, 1250, 2500 and 5000 µM of DPPH and media were collected from control and treated cells after 24 h. ELISA experiments were performed in three biological replicates. The ELISA microplate was treated with capture antibody overnight. Blocking buffer was used for 1 h at RT to remove binding sites in each well. Samples were added to wells in triplicate for 2 h at RT. Then, after washing 3–4 times with washing buffer, detection antibody (Goat Anti-Rabbit IgG H&L, Alexa Fluor^®^ 488, Abcam, product No. ab150077) with alexa fluor conjugated was used to detect PGE_2_ primary antibody bound in each well. Plates were analysed using microplate reader Infinite^®^ M1000 PRO (Tecan, Ex: 495 nm, Em: 519 nm). PGE_2_ levels were expressed RFUs (Relative Fluorescence Units). To test for the non-specific binding of the secondary antibody, samples were incubated with the secondary antibody only.

### 4.8. Statistical Analysis

All results are expressed as the mean values from three independent biological replicates with their standard errors. Statistical significances were calculated by one-way ANOVA (ANalysis Of VAriance) and Dunnett’s multiple comparison test using GraphPad Prism version 5 software. 

## 5. Conclusions

Our results describe, for the first time, the use of DPPH as an in vitro oxidising agent on human prostatic cells. The new cell-based oxidative stress model was validated as an in vitro tool for the screening of antioxidant compounds. To date, H_2_O_2_ is the most used oxidising agent in cell-based assays. The in vitro system described in the present study (PNT2 cells injured with DPPH), offers an alternative model for the study of oxidative stress and antioxidant compounds at different levels, such as sub-lethal conditions (stimulation of ROS production and inflammation) and at lethal (cytotoxic) conditions (involvement of cell death). This cell-based model can be integrated with the well-known DPPH assay, representing a novelty in the screening and identification of new antioxidant natural products. The integrated system permits to investigate cell response involved in the antioxidant effect at various levels, such as cell viability, genes, inflammatory proteins and ROS production. This combination of biochemical- and cell-based protocols can be further validated for the quick screening pipeline of a large amount of natural extracts, giving precious information on the bioactivity of samples and their mechanism of action.

## Figures and Tables

**Table 1:** Functional role of genes found up- or downregulated in PNT2 cells treated with DPPH.

GENE	Function	References
OXIDATIVE STRESS		
PRDX6	It encodes for an enzyme able to reduce hydroperoxides, protecting cells against oxidative injuries	[28]
TXNRD1	It reduces thioredoxins and other small redox proteins, contributing to redox homoeostasis	[29,30]
SOD1	It encodes for one of the three members of superoxide dismutases family, able to initiate the elimination of superoxide radicals in cells	[31]
NOX5	It encodes for an oxidase involved in the production of ROS	[30]
TPO	It is a thyroid specific gene connected with peroxiredoxin gene family members and SODs genes, involved in the reduction of deleterious effects of ROS	[32,33]
EPHX2	It belongs to epoxide hydrolase family and silenced gene prevents prooxidants-induced cell damages	[34]
MT3	It reacts with oxidising agents, reducing levels of these harmful molecules	[35]
INFLAMMATION		
MPO	It is implicated in the induction of inflammatory process and is connected with PTGS1 gene	[36]
PTGS1	It is involved in the synthesis of prostaglandin E_2_ (PGE_2_), an inflammatory mediator with cytoprotective and antioxidant functions	[37]
ATOX1	It contributes to inflammatory response and to defend cells against many free radical species	[38]
MBL2	It interconnects oxidative stress, inflammation and activation of immune system	[39]
SURVIVAL/CELL DEATH		
BNIP3	It controls survival/death mechanisms; it can promote cell survival if cell reduce oxidative stress or trigger necrosis, autophagy or apoptosis when oxidative injuries compromise cell functions	[40]
SLC7A11	It is responsible of glutathione biosynthesis and participates of protection mechanisms against oxidative injuries and regulation of ferroptotic cell death	[41]
MPV17	It activates peroxisomal ROS metabolism; its activity is essential to avoid mitochondrial dysfunction and cell death	[42]
SIRT2	It protects mitochondria and DNA from oxidative damages; if oxidative stress causes severe damages, it act as pro-apoptotic factor	[43]

## Supplementary Materials

Supplementary materials can be found at https://www.mdpi.com/1422-0067/21/22/8707/s1.
