# Supplementary material for: New In Vitro Model of Oxidative Stress: Human Prostate Cells Injured with 2,2-diphenyl-1-picrylhydrazyl (DPPH) for the Screening of Antioxidants
Source: Int J Mol Sci. 2020 Nov 18;21(22):8707. doi: 10.3390/ijms21228707 (PMC7698958; doi:10.3390/ijms21228707)
Supplement: Supplementary file 1 [file ijms-21-08707-s001.zip › ijms-993424-supplementary.docx]

**Supplementary Materials**

**Table S1**. List of all oxidative stress genes analysed, grouped for gene family (some genes are involved in more than one antioxidant mechanism). Genes in bold were found differently expressed with respect to the control.

| GENE SUPERFAMILY | GENE SUBFAMILY | GENES ANALYSED |
| --- | --- | --- |
| Antioxidants | Glutathione Peroxidases (GPx) | **GPX1**, GPX2, GPX3, GPX4, GPX5, GSTP1, GSTZ1. |
|  | Peroxiredoxins (TPx) | PRDX1, PRDX2, PRDX3, PRDX4, **PRDX5**, **PRDX6** (AOP2). |
|  | Other Peroxidases | CAT, **CYBB**, CYGB, DUOX1, DUOX2, EPX, LPO, **MPO**, **PTGS1** (COX1), PTGS2 (COX2), TPO, TTN. |
|  | Other Antioxidants | ALB, GSR, **MT3**, VIMP, SOD1, SOD3, SRXN1, **TXNRD1**, TXNRD2. |
| Reactive Oxygen Species Metabolism | Superoxide Dismutases (SOD) | **SOD1**, SOD2, SOD3. |
|  | Other Superoxide Metabolism Genes | ALOX12, CCS, DUOX1, DUOX2, MT3, NCF1, NCF2, NOS2 (INOS), NOX4, **NOX5**, UCP2. |
|  | Other Reactive Oxygen Species Metabolism Genes | AOX1, **BNIP3**, **EPHX2**, **MPV17**, SFTPD. |
|  | Oxidative Stress Responsive Genes | **APOE**, **ATOX1**, CAT, CCL5 (RANTES), CYGB, DHCR24, DUOX1, DUOX2, DUSP1 (PTPN16), EPX, FOXM1, FTH1, GCLC, GCLM, GPX1, GPX2, GPX3, GPX4, GPX5, GSR, GSS, HMOX1, HSPA1A (HSP70−1A), KRT1, LPO, **MBL2**, MPO, **MSRA**, NQO1, NUDT1, PDLIM1, PRDX2, PRDX5, PRDX6 (AOP2), PRNP, RNF7, VIMP, **SEPP1**, **SIRT2**, **SLC7A11,** SOD1, SOD2, SQSTM1, SRXN1, **TPO,** TTN, TXN, TXNRD1, TXNRD2. |

**Table S2**. List of the 21 genes involved in oxidative stress that showed a significant variation of gene expression (positive fold change values are reported in green, negative ones in red).

|  |  |  |
| --- | --- | --- |
| GENE | **Description** | **PNT2 + DPPH** |
| GPX1 | Glutathione peroxidase 1 | **−2.03** |
| PRDX5 | Peroxiredoxin 5 | **−2.12** |
| CYBB | Cytochrome b-245, beta polypeptide | **−2.29** |
| PRDX6 | Peroxiredoxin 6 | **2.12** |
| MPO | Myeloperoxidase | **2.77** |
| PTGS1 | Prostaglandin−endoperoxide synthase 1 | **3.07** |
| MT3 | Metallothionein 3 | **7.95** |
| TXNRD1 | Thioredoxin reductase 1 | **2.08** |
| EPHX2 | Epoxide hydrolase 2, cytoplasmic | **−2.19** |
| APOE | Apolipoprotein E | **−5.33** |
| MSRA | Methionine sulfoxide reductase A | **−3.05** |
| SEPP1 | Selenoprotein P, plasma, 1 | **−2.15** |
| SOD1 | Superoxide dismutase 1, soluble | **4.54** |
| NOX5 | NADPH oxidase, EF−hand calcium binding domain 5 | **2.04** |
| BNIP3 | BCL2/adenovirus E1B 19kDa interacting protein 3 | **3.80** |
| MPV17 | MpV17 mitochondrial inner membrane protein | **7.31** |
| ATOX1 | ATX1 antioxidant protein 1 homolog (yeast) | **3.56** |
| MBL2 | Mannose−binding lectin (protein C) 2, soluble | **4.80** |
| SIRT2 | Sirtuin 2 | **6.95** |
| SLC7A11 | Solute carrier family 7, member 11 | **2.24** |
| TPO | Thyroid peroxidase | **5.24** |
